# TB case detection: can we remain passive while the process is active?

**Published:** 2012-03-19

**Authors:** Markos Abebe, Mark Doherty, Liya Wassie, Abebech Demissie, Adane Mihret, Howard Engers, Abraham Aseffa

**Affiliations:** 1 Armauer Hansen Research Institute, Addis Ababa, Ethiopia; 2 Statens Serum Institute, Copenhagen, Denmark

**Keywords:** Case detection, Tuberculosis, DOTS

## Abstract

TB remains a major public health problem despite all the efforts that have been made since it was declared a global emergency in 1993. Different strategies have been implemented to curb the spread of the epidemic. Early case detection and treatment is one of the pillars of the TB control program. In 1991, WHO set targets for increasing case detection and treatment success rates to 70% and 85% respectively. Although the target of treatment success rate has been achieved, the case detection rate remains far below target at currently less than 50%. It is high time that control programs move from simple passive to a more systematic active case finding in order to accelerate TB control.

## Opinion

Tuberculosis (TB) is an aerosol-transmitted infectious disease caused by *Mycobacterium tuberculosis* (Mtb). It is estimated that one-third of the global population is infected with Mtb and 2-3 million deaths occur each year [1]. Different strategies have been employed to control TB globally including vaccination, early diagnosis and treatment of active TB disease and of latently infected individuals [2,3]. Early treatment of active cases depends upon efficient and timely diagnosis, which is not easy - at least in developing countries - based on the current diagnostic tools and strategies. Treatment of latently infected individuals reduces the chances of subsequent reactivation of disease, but has frequent side effects, making it impractical in TB-endemic countries given the high proportion of latently infected individuals, the possible risk of re-infection [4,5] and the huge cost it would incur. As a result of such reasons, the control of TB remains a major challenge. Consequently, the world is facing a large number of new cases every year – in the range of 8-10 million [6]. This means that transmission of the disease remains unbroken, because theoretically, a single untreated TB patient can infect (on average) 10-15 people per year [7].

In Ethiopia, the incidence of all forms of TB and of smear positive TB is 341 and 152 per 100,000 population respectively. On top of this burden, the emergence of MDR and XDR TB is becoming a real threat. Based on few studies, the proportion of MDR-TB is 1.6% and 11.8% among new cases and retreatment cases respectively [8].

The TB control program in Ethiopia introduced DOTS as a pilot programme in 1992 and DOTS geographical coverage reached 100% in 2006 (http://www.stoptb.org/assets/documents/countries/acsm/Ethiopia.pdf). However, the case detection rate is still below 50% [9].

The purpose of this article is to comment on the existing TB case finding strategy within the framework of DOTS because, the most efficient method of preventing transmission is identification – through early case detection, diagnosis and treatment of pulmonary TB patients; who are the most infectious source [10].

Hailed as a major step towards the goal of controlling TB, DOTS was introduced 20 years ago as an instrument to tackle the problem of TB globally. DOTS is generally an inexpensive and highly effective means of treating patients already infected with TB, thus preventing new infections and the development of drug resistance. Accordingly, 182 countries had implemented the DOTS strategy by the end of 2003. Through the DOTS program, 1.8 million new TB cases were picked up through lab testing in 2003; which gives a case detection rate of 45% which, while an improvement over earlier efforts, is still well below what WHO had aimed to achieve i.e., a 70% case detection by 2005. Moreover, the U.N. Millennium Development Goals include targets to “halve the 1990 TB prevalence and death rates by 2015” (http://www.tbalert.org/worldwide/DOTS.php). Can we hope to reach this goal in the remaining 4 years? This will be challenging because, nearly twenty years after the start of WHO′s DOTS strategy, tuberculosis remains a major global health problem and though the DOTS technical package has improved overall treatment success, DOTS expansion has had little or no effect on case detection [11]. It appears that we will repeat the failure of the 1991targets set by the WHO Assembly in which two targets for TB control were established i.e., 70% of case detection rate and 85% cure rate by the year 2000 - this too was not achieved [12].

One of the main reasons for this is lack of effective case detection strategy as an essential arm of the TB control program. Although DOTS has been successful in bringing about an 18% increase in treatment success rate, bringing us close to the target of 85% success, we have made little real progress on case detection [11]. This shows that control programs need to revise their strategy to increase the case detection rate. The existing WHO strategy is to employ passive case finding as opposed to “more resource-intensive” active case finding [10,13,14]. The difference between these two strategies is very wide. In the case of active case detection, the health care workers actively search for the cases and help them to get treated as early as possible while with passive case detection, it is expected that the patients themselves will come to the health facilities when they become ill. The latter strategy depends on high awareness and early health care seeking behaviour of a given community; which is often weak in most developing countries. The weakness of this strategy is that if infectious patients do not come early or at all, or come only when severely ill, they remain a source of new cases among the community – for months, or sometimes years.

Current TB control policy emphasizes case finding through sputum smear microscopy for patients who self-report to primary health centers [13,14]. However, this means that the cycle of infection is not being broken-it is evident that the risk of developing active TB among symptomatic household contacts of index cases identified this way is very high. Studies by Murray et al showed that the TB prevalence detected through combined active and passive case finding among household contacts was much higher than with passive case finding alone [13]. Based on this finding they suggested contact tracing as a powerful means of improving case detection rates for active TB disease. A study by Zachariah et al also revealed that prevalence of TB by passive case finding among household contacts was significantly lower than with active case finding [15]. Other studies have also recommended active case finding through contact tracing and targeted screening of high risk groups [3,10,13,15–18]. A study by Yimer et al has shown that through active case finding: among the 47,478 individuals living in households that were screened, 1006 TB suspects and 38 cases were detected [19]. This resulted in a rate of smear-positive TB, of 80 per 100,000 population – far higher than the official estimate, based on passive surveillance. The ratio of active vs. passive case detection was 2.5:1, indicating 2.5 undetected TB cases in the community for every smear-positive TB case receiving treatment during the survey period. Like so many, this study revealed a very high proportion of TB cases goes undiagnosed until they eventually turn up at a health facility-which then becomes diagnosed. This indicates that the potential for a large infectious pool and significant transmission of TB in the community is high. Therefore, the expansion of diagnostic facilities and the active involvement of health extension workers is necessary to expedite early detection, timely referral and treatment of TB [19,20].

India′s Revised National TB Control Programme recommends screening of all household contacts of smear-positive pulmonary tuberculosis cases for tuberculosis (TB) disease and provision of isoniazid preventive therapy (IPT)[16]. They recommended that contact tracing could increase the identification of active pulmonary TB case through tracking down the household contacts of newly diagnosed TB cases [21]. Mathematical models also suggested that increasing TB detection and cure rates by a more proactive strategy would be the most effective way to reduce TB incidence and burden [22].

A recent review has listed these proactive strategies as including tuberculin surveys (though not advised in all settings), mass radiography, house-to-house surveys and systematic outpatient screening [10]. Lonnoroth et al also suggested a framework for identification of relevant entry points for improved early case detection through identification of contacts (children, other risk groups, all household, workplace), clinical risk groups (HIV, previous TB, malnourished, smokers, diabetics, drug abusers) and risk populations (prisons, urban slums, poor areas, migrants, workplace, elderly) [12,23,24]. The choice of which approach or combination of approaches to use should be guided by the local epidemiology and available resources [18].

In a longitudinal study (VACSEL/VACSIS) conducted in Butajira, Ethiopia, we recruited a total of 157 subjects of which 41 were newly diagnosed smear positive Pulmonary Tuberculosis (PTB) patients and 116 were household contacts of those TB patients. We followed these individuals for up to 3 years. Then we studied if those household contacts had developed active TB or not. Contacts at the time of entry to the study were without any signs and symptoms for TB (AFB negative, normal chest x-ray, no previous TB history) but they had been living with PTB patients for at least 6 months.

The follow up study revealed that 13.8% (16 out of 116 HC) of the household contacts had developed active TB (PTB or EPTB) and were on treatment at Butajira Hospital (unpublished data).

According to the literature, it is estimated that 30% of exposed individuals get infected, of whom 10% will develop disease within 2 years of infection, while 90% remain latently-infected with a subsequent 10% risk of reactivation throughout their life time [25–27]. At first glance, if we compare our findings with the assumption of 30% infection rate and 10% progression to active TB, our observation is significantly higher - 16 out of 116 contacts in this study have developed TB, which is 4 fold higher than the global assumption. However, the numbers reported here are for household contacts of sputum-positive index cases – a particularly high-risk group and the elevated risk is comparable to that reported elsewhere [28]. Particularly when one considers the limitations of the study: because of consent requirements (we only included household contacts into the study following consent, aged between 15 and 65), we did not include all household contacts of the index case, making the denominator smaller. On average we recruited 3 household contacts for each index case. However, this data certainly supports the strategy to actively find early cases of TB among household contacts of TB cases.

## Conclusion and way forward

One may argue that active case detection is more demanding in terms of requirements for improved health care facility, trained personnel and budget to cover expenses but the cumulative and longstanding effect could be the breaking of the cycle of transmission as well as reducing MDR-TB [29]. One of the main reasons for countries to pursue passive case detection instead is lack of healthcare resources. This calls for studies on cost effective approaches of active case detection rather than ruling it out. The evidence of the last two decades shows us clearly that a high treatment success rate is of little help unless it is paired with a high rate of case detection. Our own work, and that of others suggests that one way to start addressing this problem (the need for active case detection vs. the lack of resources to pursue this approach) is to implement an intermediate strategy: targeted testing in high risk groups [16,30,31]. Takele et al., (manuscript submitted) reported that identifying geographical clusters of high prevalent areas based on data obtained through passive case finding would help to focus and direct available resources to conduct active case finding.

In our study, when TB patients came to the hospital, they were asked to bring their household contacts and both were given detailed examinations. This should be encouraged and introduced in the current TB control program in order to capture suspected cases: the high number of otherwise-unidentified cases found among close contacts suggests it would be a very cost-effective strategy. It is also crucial to increase community awareness about TB transmission, symptoms etc to enhance their health seeking behaviour and encourage people to show up to the nearby health institutions for any suspicion of TB. At the same time it is worth teaching the community that TB is not always linked to HIV, which has stigmatizing effect and may be a stumbling block inhibiting TB suspects from coming to health centres.

Some of the five key components of the DOTS strategy; which were endorsed in 1994 need to be revised in light of the current situation and based on the significance of their outcome. Because for example, strategy No. 2 [14] recommends “Case detection by sputum smear microscopy among symptomatic patients self reporting to health facilities” this implicitly favours passive case detection [11]. Given that passive case detection has proven to be insufficient, some countries have already introduced active case finding in their programs while the WHO guideline still remains the same.

The most powerful intervention to reduce transmission of TB is to reduce the diagnostic delays (http://www.who.int/docstore/gtb, http://www.aidsmap.com/cms1199985.aspx) and this can be achieved better through active case finding, increasing the proportion of infectious cases identified, and ensuring earlier diagnosis [32,33] so that the duration of infectiousness before effective chemotherapy is initiated [34] would be shortened. Overall, the DOTS strategy has been a useful tool in that, DOTS patients who are taking their drugs in front of health care workers appears to improve outcomes-but it addresses only those who show up themselves to the health services.

Therefore, taking as an opportunity the current progress in the expansion of health extension workers (HEW) and community participation at the kebele level here in Ethiopia, it would be possible to establish an updated strategy for case detection [20]. Of interest is that in the 2008 Ethiopian NTLP manual, “active case detection” (through contact tracing) has been introduced. But more can be done than contact tracing alone by involving HEWs to identify individuals coughing for 2 weeks (out-patient symptom screening) [10] and bringing them to the nearby health facility to be tested for TB [20]. The National TB control Program, on top of passive and enhanced case finding, should direct its case finding strategy towards active case finding through: contact tracing [16], out-patient symptom screening using 2 weeks cough as tracker [10], targeted screening of high risk groups [12] and identifying geographical clusters of high burden areas (Takele et al, manuscript submitted) and conduct active case finding in those clusters.
